# Natural Ecosystem Surrounding a Conventional Banana Crop Improves Plant Health and Fruit Quality

**DOI:** 10.3389/fpls.2018.00759

**Published:** 2018-06-07

**Authors:** Florence P. Castelan, Victor C. Castro-Alves, Lorenzo A. Saraiva, Talita P. Nascimento, Maria F. N. S. Cálhau, Carlos T. S. Dias, Beatriz R. Cordenunsi-Lysenko

**Affiliations:** ^1^Department of Food Science and Experimental Nutrition, School of Pharmaceutical Sciences, University of São Paulo, São Paulo, Brazil; ^2^Food Research Center, Research, Innovation and Dissemination Centers, São Paulo Research Foundation, São Paulo, Brazil; ^3^Department of Exact Sciences, Luiz de Queiroz College of Agriculture, University of São Paulo, Piracicaba, Brazil; ^4^Food and Nutrition Research Center (NAPAN), University of São Paulo, São Paulo, Brazil

**Keywords:** Atlantic forest, banana, biodiversity, carbohydrate, greenlife, plant growth regulators, postharvest, ripening

## Abstract

Natural ecosystems near agricultural landscapes may provide rich environments for growing crops. However, the effect of a natural ecosystem on crop health and fruit quality is poorly understood. In the present study, it was investigated whether the presence of a natural ecosystem surrounding a crop area influences banana plant health and fruit postharvest behavior. Plants from two conventional banana crop areas with identical planting time and cultural practices were used; the only difference between banana crop areas is that one area was surrounded by a natural forest (Atlantic forest) fragment (Near-NF), while the other area was inserted at the center of a conventional banana crop (Distant-NF). Results showed that bananas harvested from Near-NF showed higher greenlife and a more homogeneous profile during ripening compared to fruits harvested from Distant-NF. Differences in quality parameters including greenlife, carbohydrate profile, and pulp firmness between fruits harvested from Near-NF and Distant-NF are explained, at least partly, by differences in the balance of plant growth regulators (indole-3-acetic acid and abscisic acid) in bananas during ripening. Furthermore, plants from Near-NF showed a lower severity index of black leaf streak disease (BLSD) and higher levels of phenolic compounds in leaves compared to plants from Distant-NF. Together, the results provide additional evidence on how the maintenance of natural ecosystems near conventional crop areas could be a promising tool to improve plant health and fruit quality.

## Highlights

In this “proof of principle” study, an innovative approach isolated natural biodiversity as the only variable between two banana crop areas, highlighting biodiversity benefits on crop health and fruit quality.

## Introduction

The exchange between organisms, materials, and energy occurs in between different ecosystems. Hence, agricultural areas should consider mosaics of habitats composed not only of crops but also forestry, fragments, pastures, and fallow fields nearby the crops (Altieri and Nicholls, 2003). Natural ecosystems nearby agricultural landscapes can provide richer environments for growing crops by enhancing natural control of pests (Gardner et al., 2009) and diseases (Kageyama et al., 2002; Tomas et al., 2009; Claflin et al., 2017), nutrient cycling, erosion control, and carbon sequestration (Jarvis et al., 2007). Thus, organic crop farming and agro-environmental management within and around production areas could increase crop resilience and reinforce food security against climate change and resource scarcity (Batáry et al., 2010; Frison et al., 2011; Gonthier et al., 2014). Although the mechanism through which the natural biodiversity influences crop health and fruit quality is not well-understood, American producers have been managing non-production areas through “farmscaping”, thereby enhancing biodiversity that leads to multiple ecosystem services. (Smukler et al., 2010).

In the past few decades, several studies compared organic/sustainable and conventional crop system in terms of plant health and fruit quality (Castro et al., 2015; Pertuzatti et al., 2015; de Oliveira et al., 2017). Despite issues related to sampling standardization (Bourn and Prescott, 2002) and field variables (Seufert et al., 2012; Connor, 2013), most of studies showed that organic food crops appear to grow in a healthier manner compared to conventional crops. Fruits from organic/sustainable crops had higher levels of bioactive compounds (Hurtado-Barroso et al., 2017; Ren et al., 2017), whereas fruits from conventional crops had higher levels of nitrogenous compounds (Bourn and Prescott, 2002). Moreover, fruit from organic/sustainable crops had longer shelf life and delayed senescence while attached to the plant (Neelam et al., 2008; Reganold et al., 2010). Thus, although a reduced yield performance of organic crops compared to conventional crops is discussed (De Ponti et al., 2012; Seufert et al., 2012; Connor, 2013), organic/sustainable crops are commonly associated with increased plant health and fruit quality and if these crops were based on a diversified system, the yield performance gap appears to reduce, suggesting that this gap is a matter of management investment in agrobiodiversity improvement (Ponisio et al., 2015).

Thus, if an enhanced biodiversity plays a key role in the functioning of agroecosystems, information about the effects of biodiversity on crop health and food quality should be addressed, especially if the effects of biodiversity were evaluated exclusively rather than within a larger set of cultural practices. Therefore, with this challenge in mind, this study investigated whether the natural biodiversity surrounding influences banana (*Musa acuminate*, Cavendish subgroup, cv. Nanicão) plant health and fruit quality by investigating two areas of conventional banana crops with identical planting time and cultural practices. However, one area (Near-NF) was surrounded by a natural forest fragment of Atlantic Forest – the most damaged Brazilian biome and one of the top hotspots worldwide (Myers et al., 2000) – while the other area (Distant-NF) was inserted at the center of a conventional banana crop.

Banana is a key crop for Brazilian fruit production (Saraiva et al., 2018) and its fruit development is dependent on accumulated degree-days (DD) starting from plant flowering until the harvest period (Blake and Peacok, 1971; Ganry and Meyer, 1975; Jullien et al., 2008). Banana fruits from cv. Nanicão are harvested in a pre-climacteric stage at approximately 900 DD in subtropical conditions to be further transported and commercialized. The time between the harvest at the pre-climacteric stage until the onset of ripening, which is characterized by the pronouncement on endogenous ethylene production (Pech et al., 2012), is defined as greenlife, which is an important parameter of fruit quality since it is directly related to fruit shelf life. In addition to ethylene, other plant growth regulators including indole-3-acetic acid (IAA) and abscisic acid (ABA) are associated with regulation of fruit greenlife and conversion of starch into soluble sugars (Jiang et al., 2000; Purgatto et al., 2001, 2002), thereby regulating fruit sweetening and softening, which are quality attributes of ripe banana fruits. Increased IAA levels in banana fruits are associated with delayed degradation of starch, higher greenlife, and increased shelf life, whereas increased ABA levels play an opposite role, reducing climacteric fruit greenlife and accelerating fruit senescence (Jia et al., 2016). Thus, possible differences between the profile of these plant grown regulators during banana ripening may help to define whether a natural biodiversity surrounding a conventional banana crop influences fruit postharvest behavior and quality. In this study, bananas harvested from Near-NF and Distant-NF with same physiological age (DD) were analyzed for their profile of plant growth regulators, as well as for greenlife and carbohydrate profile and pulp firmness during ripening. Furthermore, aspects of plant health were analyzed to explore potential mechanisms through which natural biodiversity influences fruit quality.

## Materials and Methods

### Field Characterization and Plant Analysis

#### Field Experiment

**FIGURE 1:**
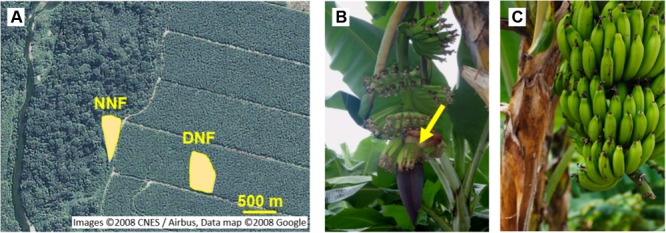
Banana experimental areas and flowering stage. **(A)** Near Natural Forest (Near-NF; 24°17′22.91″S 48° 6′24.72″W) and Distant Natural Forest (Distant-NF; 24°17′26.30″S 48° 6′17.90″W) banana areas (Images 2008 CNES/Airbus, Data map 2008 Google). Representative images of **(B)** fruits at flowering stage, characterized by the presence of male (smaller) and female (larger) flowers in the last open bract (yellow arrow), and **(C)** banana fruit at approximately 900 DD.

Field experiments were carried out using a conventional banana field with two distinct areas (*Musa acuminata*, Cavendish subgroup, cv. Nanicão): (1) Near-NF area, with 60% of the perimeter surrounded by Atlantic Forest fragment and (2) Distant-NF area, surrounded exclusively by conventional banana crops with a radius of at least 200 m (**Figure 1A**). Each area has approximately 4,000 m^2^ of flat ground and was composed of approximately 500 plants with identical planting time and receiving identical agricultural practices (chemical fertilizers, pyrethroid spraying on new bunches, carbofuran application on harvested plants, and aerial spraying of triazole and strobilurin).

#### Characterization of the Atlantic Forest Fragment

Shannon and Simpson diversity indexes (Gliessman, 2014) were determined at the beginning of the experiment using 11 collection points (1 m^2^) of the Atlantic Forest fragment surrounding the Near-NF area. A full identification of plant species from the Atlantic Forest fragment was also performed.

#### Soil

Soil samples from five different points were collected at the beginning of the field experiment and macro- and micronutrients (P, K, Ca, Mg, Al, B, S, and Cu) were analyzed (Cesar et al., 2009). Results were expressed as cmol⋅dm^-3^, mg⋅dm^-3^ or g⋅dm^-3^ for macro, C and micronutrients respectively (Supplementary Table S1). Textural classes were determined according to the Atterberg classification.

#### Plant Nutrition

Leaves from five plants per area (not the sampled plants) were collected at the beginning of flowering emission. Macro- and micronutrients from the leaves were analyzed using spectrophotometry (K, Fe, Mn, Zn, Cu, and B) or Kjeldahl (N) assay. Results were expressed as g⋅kg^-1^ and mg⋅kg^-1^ for macro- and micronutrients respectively (Supplementary Table S2).

#### Meteorological Parameters

Temperature probes (Tinytag Plus 2, TGP-4500, Gemini Data Loggers, Chichester, United Kingdom) were placed under a shelter on each experimental area. Mean daily temperature was calculated using hourly temperature readings (Supplementary Figure S1). S-LIA-M003 and S-LIB-M003 sensors (Onset HOBO Data Loggers, Bourne, United States) were placed above the banana plants to monitor photosynthetically active radiation (PAR) and global radiation respectively (Supplementary Figure S2).

#### Black Leaf Streak Disease Severity Index

Black leaf streak disease (BLSD)/black Sigatoka severity index (SI) were measured for each banana plant similar as described previously (Irish et al., 2013) at both flowering and harvest times using a scale based on the percentage of necrotic leaf tissue in all plant leaves. Since leaf emission is stopped at flowering time, the same leaves are exposed for disease during the whole flowering to harvest period. This implicates that the BLSD-SI at harvest time is a better measure of differences in disease severity compared to BLSD-SI at flowering. Results of BLSD-SI were expressed as percentage of necrosis per plant.

#### Phenolic Compounds in Leaves

Total Soluble Phenolic Compounds (TSPC) from banana leaves were extracted and quantified as described previously (Castro-Alves and Cordenunsi, 2015). Briefly, frozen and grinded banana leaves were mixed with 80% acetone and homogenized. After centrifugation, the supernatant was collected. Extraction with 80% acetone was repeated two times using the same precipitate. Resulting supernatants were combined and hexane was added to the extract. The mixture was homogenized and centrifuged to form two phases. The TSPC from the aqueous phase (bottom phase) were mixed with the Folin–Ciocalteu reagent (Sigma, St. Louis, United States) and the absorbance was read at 765 nm. Results were expressed as mg equivalent of gallic acid (GAE)⋅g^-1^ of fresh leaves.

### Fruit Sampling

For almost 2 years (2012/13), field inspections were conducted two times a month to search and monitor banana plants at the flowering stage (**Figure 1B**). The calculation of physiological age using the mean daily temperature sum (DD at 14°C threshold) during the flowering to harvest period (Ganry and Meyer, 1975) defined the harvest time. Harvest time was determined by the physiological age, predefined at 900 DD, based on previous experience, and in accordance with previous studies (Ganry and Meyer, 1975). At least three plants per area were marked according to the availability of flowering plants on the day of inspection (around 20 field inspections). In the end, eight sample collections with more than 30 plants per area in total were obtained. Differences in the ripening behavior of fruits from the Near-NF and Distant-NF area were consistent between samplings. Thus, based on the number of plants marked, and on the season, four sample collections (around 40 plants; **Table 1**) were selected for physiological and biochemical analyses. After harvest, fruits were treated with thiabendazole (Sponholz et al., 2004), weighed, measured, and stored at a controlled temperature of 20 ± 0.2°C. Analyses were carried out using fruits from the two central hands of the bunch. Fruits harvested from each plant were characterized as an individual group.

**Table 1:** Description of sample collections.

	Winter		Spring	
	W1	W2	S1	S2
Flowering date	5/8/2012	4/26/2013	9/11/2012	10/23/2012
Harvesting date	10/8/2012	10/22/2013	12/21/2012	1/21/2013
FHP (days)	154	179	101	90
Physiological age (DD)	915	905	906	909
Plants from Distant-NF (*n*)	6	5	4	5
Plants from Near-NF (*n*)	5	4	3	5

*Fruits were harvested from Near Natural Forest (Near-NF) and Distant Natural Forest (Distant-NF) banana experimental areas two times in the winter (W1 and W2) and two times in the spring (S1 and S2). FHP, flowering-to-harvest period; DD, degree-days; *n*, number of plants selected in each experimental area by sample collection.*

### Fruit Characterization

After harvest, fresh fruits were assessed throughout the entire ripening process. Samples were periodically analyzed for ethylene production, peel color, and pulp firmness and representative samples from each group were frozen at -80°C for further analysis, as illustrated in Supplementary Figure S6.

#### Ethylene Analysis and Greenlife

Ethylene production was measured daily using a gas chromatograph (GC-6890, Hewlett Packard, Stockport, United Kingdom) coupled to a flame ionization detector (GC-FID). Briefly, fruits (*n* = 3) representative of each plant were selected daily and kept in airtight glass bottles. Then, headspace samples were analyzed using a HP-PLOT Q column (30 m × 0.53 mm, 40 μm, Hewlett Packard). Helium was the carrier gas. Greenlife was determined similar as described previously (Castelan et al., 2012) as the number of days between the harvest and the initiation of the natural ripening process that is defined as the day in which the fruits reached a mean of 1.0 μL C_2_H_4_⋅kg^-1^⋅h^-1^.

#### Peel Color

Peel color of fruits analyzed for ethylene production were measured periodically using a Color Quest XE colorimeter (HunterLab, Reston, United States). Peel color (hue angle) of each fruit was expressed as the mean of six different points per fruit.

#### Pulp Firmness

Pulp firmness of fruits analyzed for ethylene production was measured periodically using a penetrometer (TA.XT2i, Stable Micro Systems, Godalmin, United Kingdom), equipped with a puncture probe (3 mm diameter). A slice (10 mm) from the center of the fruit was cut and the probe penetration was set at 6 mm. Pulp firmness of each fruit was expressed as the mean of three measurements from the same slice.

#### Starch and Soluble Sugars

Starch was enzymatically determined as described previously (Cordenunsi and Lajolo, 1995). Briefly, frozen and grinded banana pulp was soaked in 0.5 M NaOH. After neutralization with acetic acid, the starch was precipitated with 80% ethanol, separated by centrifugation, and hydrolyzed with amylase (Megazyme, Wicklow, Ireland) and amyloglucosidase (Megazyme). The resulting glucose was quantified enzymatically using the glucose oxidase/peroxidase system. Soluble sugars were analyzed by high-performance anion exchange chromatography coupled with a pulse amperometric detector (HPAEC-PAD) (Shiga et al., 2011). Briefly, frozen and grinded banana pulp was extracted three times with 80% ethanol at 80°C. The ethanol was evaporated using a vacuum concentrator (Savant CS2 10A, Thermo, Waltham, MA, United States). Then, the residue was reconstituted in water, filtered, and analyzed by HPAEC-PAD ICS5000 system (Thermo) equipped with a CarboPac PA1 column (4 × 250 mm, Thermo) using 18 mM NaOH as the mobile phase. Analyses were performed in triplicate and results were expressed as g/100 g of fruit.

#### IAA and ABA Levels

Free IAA and ABA were analyzed using GC coupled with a mass spectrometer (GC-MS) similar to that described previously (Volmaro et al., 1998). Briefly, frozen and grinded banana pulp was extracted using isopropanol:acetic acid (95:5). Internal standards of IAA ([^13^C_6_]-IAA, Cambridge Isotopes, Tewksbury, United States) and ABA ([^2^H_6_]-(+)-ABA, Olchemim, Olomouc, Czechia) were added on each sample. After incubation, the mixture was centrifuged, and the supernatant was collected and concentrated under nitrogen flow. Ethyl acetate was added and the pH was adjusted to 2.5–3.5. After centrifugation, the organic phase (upper phase) was collected and the aqueous phase (bottom phase) was extracted again with using ethyl acetate. Organic phases were combined and dried using a vacuum concentrator (Thermo). The material was solubilized in methanol and methylated with trimethylsilyl diazomethane (Sigma). Then, the solution was dried, solubilized in ethyl acetate, and analyzed in a GC-MS 6890/ 5973 system (Hewlett Packard) equipped with a HP-1701 column (30 m × 0.25 mm, 0.50 μm, Hewlett Packard). IAA ions were monitored at m/z 130 and 189, corresponding to the quinolinium and the molecular ion of IAA, respectively, and at m/z 136 and 195, corresponding to equivalent ions from [^13^C_6_]-IAA. ABA ions were monitored at m/z 162 and 190 corresponding to endogenous ABA, and at m/z 166 and 194, corresponding to equivalent ions from [^2^H_6_]-(+)-ABA. Results were expressed as μg⋅g^-1^ of fruit.

### Statistical Analysis

Analyses were made following a model’s type y_ij_ = μ + A_i_ + e_ij_, where y_ij_ refers to the variable response, measured on the experimental unit of i-th area in the j-th repetition; μ is the general constant; and e_ij_ is the error of the j-th repetition for the i-th area.

For each assessed plant (biological replicate), three subsampling were carried out for each parameter analyzed, except for BLSD-SI, which is the assessment of the total necrotic area in leaves from a plant and therefore only one measure per plant was performed at flowering and harvest period. Hence, we have 37 observations – plants – in both experimental areas (**Table 1**) for all fruit parameters, which results in a total degree of freedom of 110 and 109 on the degree of freedom on the experimental error. Moreover, all assumptions of variance analysis were determined and checked (homogeneity of variances by Levene’s test, Normality by Shapiro–Wilk test, observation of outliers and sample size).

Results were represented as the mean ± SD of at least three independent experiments, unless otherwise stated in the figure/table legends. For BLSD-SI analysis, individual values corresponded to each plant and were analyzed as a set of plants by sample collection. Statistical analysis was performed using Oringin 8 software (OringinLab, Northampton, United States) and Prism 5 software (GraphPad, San Diego, United States). Significance was set at *p* < 0.05.

## Results

### Banana Areas Showed Similar Edaphoclimatic Conditions

Banana fruits from both the Near Natural Forest (Near-NF) and Distant Natural Forest (Distant-NF) areas (**Figure 1A**) were harvested starting from the flowering stage (**Figure 1B**) at controlled physiological age (approximately 900 DD) (**Figure 1C**). Fruits from 37 plants were harvested from two collections in the winter (W1 and W2, in subsequent years) and two collections in the spring (S1 and S2, in the same year). Description of collections are shown in **Table 1**.

Near-NF and Distant-NF areas were similar in terms of age, agricultural practices, and plant uniformity, both located on a flat ground. Nevertheless, soil and foliar analysis were performed and meteorological factors were monitored. No toxic and deficient levels of macro- and micronutrients were observed on soil fertility (Supplementary Table S1) and foliar analysis (Supplementary Table S2). Additionally, air temperature (Supplementary Figure S1) and global and photosynthetically active (PAR) radiation showed the same pattern for Near-NF and Distant-NF areas (Supplementary Figure S2), thereby ruling out the possibility of shading by the forest fragment in the Near-NF area. Moreover, the Shannon biodiversity index from the forest fragment was 2.7, which is high when compared to similar Atlantic Forest fragments on region (personal communication).

### Fruit Postharvest Behavior Is Affected by the Surrounding Biodiversity

**FIGURE 2:**
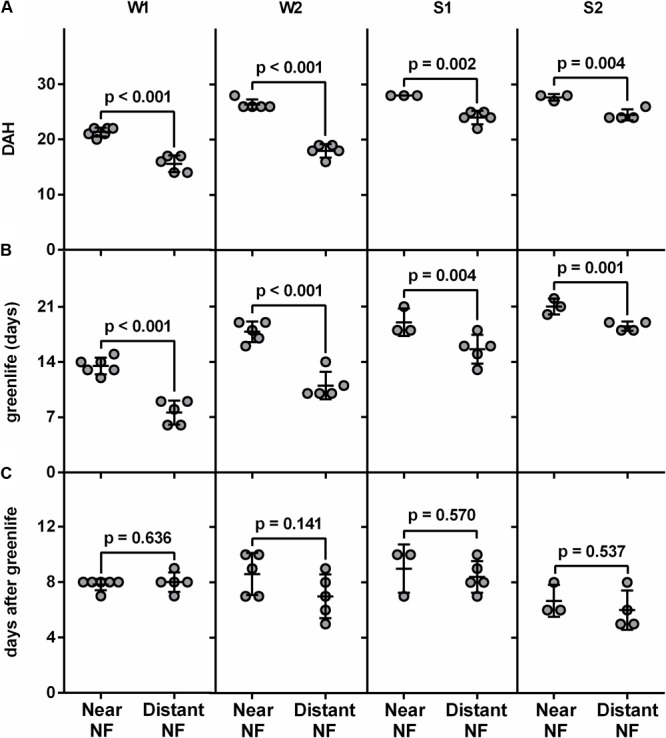
Postharvest parameters of banana fruits. **(A)** Days after harvest (DAH), **(B)** greenlife and **(C)** days after greenlife of fruits harvested from Near Natural Forest (Near-NF) and Distant Natural Forest (Distant-NF) banana areas two times in the winter (W1 and W2) and two times in the spring (S1 and S2). The end of greenlife was determined when ethylene production by the fruits reached 1.0 μL kg^-1^h^-1^. Each point represents the value of fruits from a single plant. Each bar represents the mean ± SD. The number above the bar represents *p* value (Student’s *t*-test).

After harvest at the same physiological age, fruits from each plant were monitored periodically for ethylene production, peel color and pulp firmness until full ripening (completely yellow with black spots). Interestingly, in both the winter (Supplementary Figure S3) and the spring (Supplementary Figure S4) collections, fruits harvested from the Near-NF took a longer time to achieve full ripening compared to fruits harvested from the Distant-NF area (**Figure 2A**). These differences were related to the greenlife of the fruits (**Figure 2B**) since once endogenous ethylene production was triggered, no differences in days after green-life were observed between fruits harvested from the Near-NF and Distant-NF areas (**Figure 2C**). Notably, although marked changes were observed on pulp firmness and peel color after the end of greenlife of fruits from the Near-NF and Distant-NF areas, a surprising homogeneity was observed in fruits from Near-NF area, in which all the plants seemed to develop fruits with a similar ripening profile.

**FIGURE 3:**
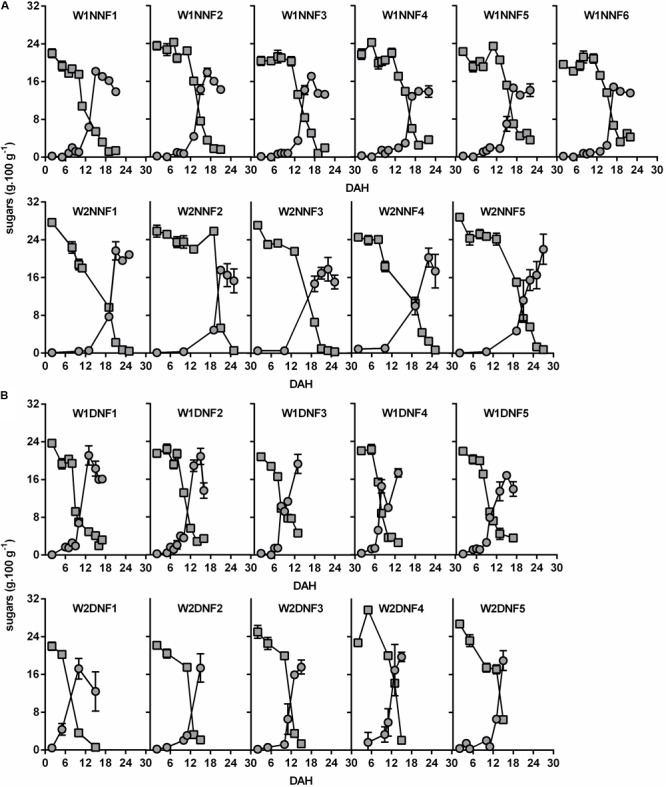
Starch (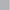) and soluble sugars (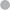) contents of banana fruit pulp during ripening. Fruits were collected two times in the winter (W1 and W2) and harvested from **(A)** Near Natural Forest (Near-NF; 11 plants) and **(B)** Distant Natural Forest (Distant-NF; 10 plants) banana experimental areas. DAH, days after harvest. Each graph represents values from fruits of a single plant. The group of fruits from a single plant was coded according to the time of collection (W1/W2), area (Near-NF: NNF/Distant-NF: DNF) and number of plants (1–6). Each point represents the mean ± SD (*n* = 3).

Focusing on the winter replicates (W1 and W2), carbohydrate analysis revealed that fruit pulp harvested from Near-NF (**Figure 3A**) and Distant-NF (**Figure 3B**) areas exhibited similar contents of starch and soluble sugars, but the beginning of the conversion of starch into soluble sugars occurred earlier in fruits from the Distant-NF area (8.7 ± 2.6 DAH) compared to fruits from Near-NF area (13.7 ± 3.7 DAH). Once again, profiles of fruits harvested from the Near-NF area seemed to be more homogeneous compared to fruits from Distant-NF area.

**FIGURE 4:**
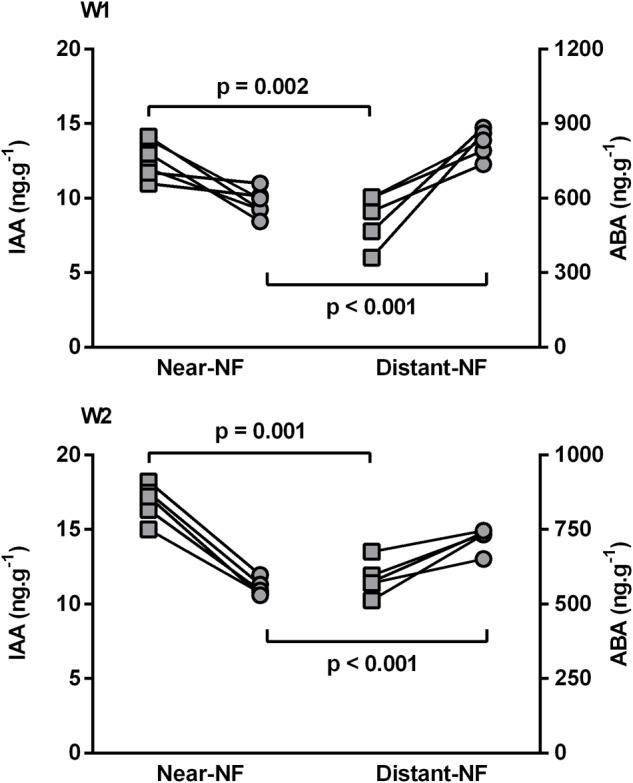
Initial levels (2 DAH) of free indole-3-acetic acid (IAA) (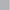) and abscisic acid (ABA) (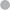) of banana fruit pulp. Fruits were harvested from Near Natural Forest (Near-NF; 11 plants) and Distant Natural Forest (Distant-NF; 10 plants) banana experimental areas collected two times in the winter (W1 and W2). Each point represents the mean value (*n* = 3) from a single plant, and *p* value was calculated using Student’s *t*-test.

The content of free IAA and ABA on fruit pulp was also analyzed. The profile of these plant growth regulators during ripening seems to have evolved in an analogous way on fruits from Near-NF and Distant-NF area. As shown in Supplementary Figure S5, free IAA decreased during ripening. On the other hand, free ABA increased during the preclimacteric phase and decreased during the climacteric and postclimacteric phases, thus exhibiting a peak before the increase in ethylene production. However, after harvest (2 DAH), fruits from the Near-NF area showed significantly higher amounts of free IAA (W1: 30.5%, *p* = 0.002; W2: 31.9%, *p* = 0.001) and significantly lower amounts of free ABA (W1/W2: 32.9%/29.4%, *p* < 0.001) compared to fruits from Distant-NF area (**Figure 4**).

### Crop Surrounding Disturb Plant Health

**FIGURE 5:**
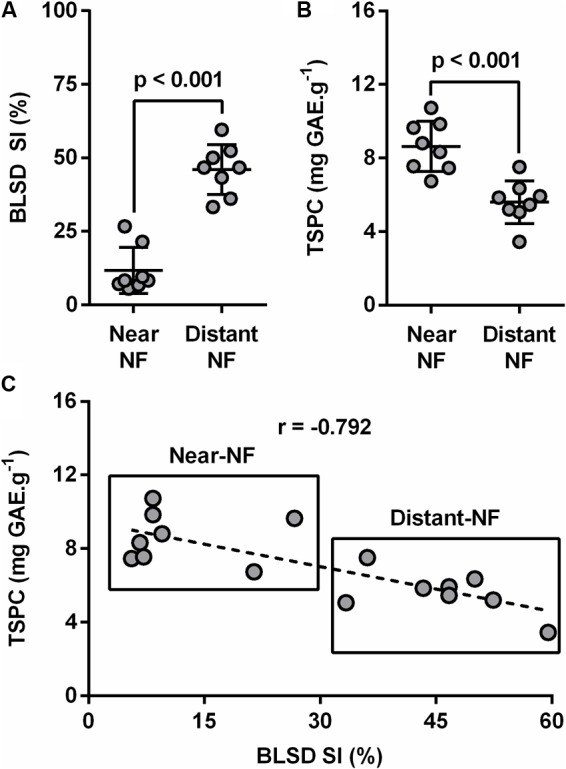
Black leaf streak disease (BLSD) severity index (SI) of banana plants and total soluble phenolic compounds (TSPC) of banana leaves. **(A)** BLSD SI, **(B)** TSPC, and **(C)** correlation between mean values of plants BLSD SI and their leaves’ TSPC. Near-NF, Near Native Forest area; Distant-NF, Distant Native Forest area. Each bar represents the mean ± SD (*n* = 8). Number above bar represents *p* value (Student’s *t*-test). Correlation (plants BLSD SI × leaves TSPC) of samples was measured using the Pearson product–moment correlation (*r*).

Black leaf streak disease severity index (BLSD-SI) was measured at both flowering and harvesting time in all plant leaves. Results suggested healthier banana plants from the Near-NF area compared to plants from Distant-NF area, especially at harvest time (**Figure 5A**). Furthermore, plant leaves from Near-NF area showed significantly higher levels (35.1%, *p* < 0.001) of TSPC (8.6 ± 1.4 mg GAE⋅g^-1^) compared to leaves of banana plants from the Distant-NF area (5.6 ± 1.1 mg GAE⋅g^-1^) (**Figure 5B**). Finally, BLSD-SI exhibited a strong and negative correlation with levels of TSPC (*r* = -0.792) at the harvest stage of banana plants from both Near-NF and Distant-NF areas (**Figure 5C**).

## Discussion

Using an innovative experimental approach, this work explored whether the proximity to the natural biodiversity of the Atlantic Forest influences the postharvest behavior of banana fruit, as well as some aspects of crop health. Previous studies have already evaluated the effects of natural biodiversity on plant health and fruit quality by comparison between crops with distinct agricultural systems/practices (Seidel et al., 2017; Fess and Benedito, 2018). However, in this study, biodiversity effects in a conventional banana field were isolated by comparing two conventional banana crop areas (Near-NF and Distant-NF; **Figure 1**) with the same age and agricultural practice. The only difference between the Near-NF and Distant-NF was that Near-NF area was surrounded on 60% of its perimeter by the natural biodiversity (an Atlantic Forest fragment), whereas Distant-NF was surrounded exclusively by banana crops.

Biochemical analysis and evaluation of postharvest parameters revealed that banana fruits from the Near-NF ripened later (higher greenlife) and came from healthier plants with lower BLSD-SI. Greenlife is an important feature of banana fruits not only to commercial aspects, but also to physiological aspects, since greenlife is influenced by the physiological age of banana fruit at harvest. Fruits harvested at higher physiological age (e.g., higher DD) had lower greenlife compared to fruits harvested at lower DD (Jullien et al., 2008). However, harmful stresses during the developmental stage of fruits can reduce fruit greenlife irrespective of DD, inducing premature ripening (Chillet et al., 2009; Castelan et al., 2012). The reduction of fruit greenlife because of biotic/abiotic stress can be considered as an adaptive behavior of the plant, which may accelerate its physiological processes to get best chances to the propagation of its next generation. In this study, “rushed” plants from the Distant-NF had lower TSPC levels on green leaf tissue compared to plants from Near-NF. TSPC levels were strongly and negatively correlated with BLSD-SI, which is in agreement with previous studies, which found that reduced TSPC levels in banana leaves are associated to increased plant susceptibility to fungal diseases, such as crown rot (Ewané et al., 2012) and BLSD (Ncho et al., 2016). Surprisingly, BLSD increases TSPC levels on the necrotic area of infected tissue (Sanchez-garcía et al., 2013); however, increased TSPC levels on the necrotic area of leaves infected by BLSD is related to a defense-related mechanism to fungal infection rather than a signal of increased resistance (Meena et al., 2016). Thus, necrotic areas from leaf tissue were avoided on the samplings used in the present study. Furthermore, a recent study showed that increased diversity of plants species is associated with increased TSPC in soil organic matter (El Moujahid et al., 2017). Thus, results suggest that the natural biodiversity surrounding may enhance phenolic compounds in soil organic matter and therefore increases TSPC levels in leaves of plants from Near-NF. Although further study is needed to confirm this hypothesis, results strongly suggests that the natural biodiversity surrounding appears to enhance TSPC levels in plant leaves from Near-NF, thereby reducing plant susceptibility to BLSD (Kulbat, 2016).

The triggering of banana ripening occurs through a complex cascade of plant growth regulators. Ethylene has long been recognized as the main regulator in the ripening of climacteric fruits including bananas (Liu et al., 1999); however, IAA and ABA also play crucial roles in banana fruit ripening (Purgatto et al., 2002; Ghosh et al., 2016). ABA induces ripening by increasing respiration rates and ethylene production, thereby inducing changes in peel color and fruit firmness during ripening of climacteric fruits (Jiang et al., 2000; Lohani et al., 2004; Zhang et al., 2009). On the other hand, banana fruit infiltrated with IAA have delayed ripening, reduced β-amylase expression and activity, and therefore delayed starch degradation and sucrose formation (Purgatto et al., 2001). Moreover, the conversion of starch into soluble sugars which promotes banana pulp sweetening and softening (Mota et al., 1997; Shiga et al., 2011) appears to be associated with a decrease in fruit IAA levels (Purgatto et al., 2002). IAA and ABA are also key plant growth regulators that impact the perception, signaling, and acclimation of plants to environmental stress (Bostock et al., 2014). Ethylene also induces the expression of several genes related to stress/defense during ripening (Kesari et al., 2010; Iqbal et al., 2017). In this study, green fruits (2 DPC) from Distant-NF showed lower free IAA levels and higher free ABA levels compared to fruits from the Near-NF. These results are in agreement with our previous study (Castelan et al., 2012), which showed that reduction of greenlife resulting from biotic stress during fruit developmental stage is associated with reduced IAA levels after harvest. Notably, these reduced IAA levels is also observed in fruits with advanced physiological age (Saraiva et al., 2013). Therefore, although fruits from Near-NF and Distant-NF were harvested at the same DD and were subjected to the same agricultural practices, fruits harvested from Distant-NF appear to be in an advanced physiological state compared to from Near-NF. In summary, we have developed a successful experimental approach for evaluating exclusively the effects of biodiversity on a crop area. Fruits harvested from Distant-NF have lower IAA and higher ABA levels compared to fruits from Near-NF. These differences in plant growth regulators profile are associated with accelerated physiological process of fruit from Distant-NF, which results in lower greenlife and faster senescence compared to fruits from Near-NF. Furthermore, the higher BLSD-SI in plants from Distant-NF can be understood as both an effect and a cause of stress responses. Lower levels of TSPC were found in the green leaf tissue of plants from Distant-NF, which suggests a disadvantage of plants from this area in the interaction with the fungus responsible for BLSD (*Mycosphaerella fijiensis*) compared to plants from Near-NF.

The results emphasize that the design of a crop area should consider the maintenance of natural biodiversity as suggested previously (Fahrig et al., 2015). Although biodiversity effects on fruit quality were never assessed using the design developed for the present study, the benefits resulting from the biodiversity improvement and heterogeneity at landscape-scale was already know to have positive effects on the biological control of pests and diseases (Bosem Baillod et al., 2017; Claflin et al., 2017), and weed management (Colbach et al., 2017). These positive effects that are driven by the improvement of biodiversity are also a key strategy of integrated crop-livestock systems (Attwood et al., 2016).

Finally, although it is difficult to predict precisely how the proximity to biodiversity could affect plant physiology, recent studies have highlighted distinct pathways through which plants are influenced by their surrounding environment (e.g., electromagnetic waves, volatile compounds, and colonization of microorganisms) (Karban, 2015). In the present study, the greatest contribution is not related to a deep understanding of crop physiology and the development of a biochemical hypotheses. Rather, this study presented an isolated approach for studying the effects of biodiversity and therefore emphasizing demonstrating that low-biodiversity landscapes surrounding a crop area do not benefit plant health and fruit quality. Thus, although ongoing studies by our group are using molecular approaches and *omics* tools to define possible mechanisms through which plants respond to their surroundings, the present “proof of principle” study demonstrates strong and consistent evidence that identifies biodiversity management as a key tool for achieving sustainable crop systems.

## Author Contributions

FC, LS, and BC-L contributed to the experimental design. FC and LS carried out the field analyses and collected samples. VC-A, TN, and MC carried out the analyses during fruit ripening. CD performed the statistical analyses. FC, VC-A, and CD did the data analysis. FC and VC-A wrote the manuscript. BC-L supervised the project and critically revised the manuscript. All authors reviewed the manuscript and have given approval to this final version.

## Conflict of Interest Statement

The authors declare that the research was conducted in the absence of any commercial or financial relationships that could be construed as a potential conflict of interest. The reviewer CL declared a shared affiliation, with no collaboration, with the authors to the handling Editor.
