# Supplementary material for: Natural Ecosystem Surrounding a Conventional Banana Crop Improves Plant Health and Fruit Quality
Source: Front Plant Sci. 2018 Jun 7;9:759. doi: 10.3389/fpls.2018.00759 (PMC6001115; doi:10.3389/fpls.2018.00759)
Supplement: Supplementary file 1 [file Presentation_1.pdf]

SUPPLEMENTARY FILE

Natural ecosystem surrounding a conventional banana crop improves plant health and fruit quality

Running title: Biodiversity improve banana crop and fruit

Florence P. Castelan[1,2], Victor C. Castro-Alves[1,2], Lorenzo A. Saraiva[1], Talita P. Nascimento[1], Maria Fernanda N. S. Cálhau1, Carlos Tadeu S. Dias[3], Beatriz Cordenunsi-Lysenko[1,2,4,*]

[1] Department of Food Science and Experimental Nutrition, School of Pharmaceutical Sciences, University of São Paulo, São Paulo, SP, Brazil
[2] Food Research Center (FoRC), CEPID-FAPESP (Research, Innovation and Dissemination Centers, São Paulo Research Foundation), São Paulo, SP, Brazil
[3] Department of Exact Sciences, Luiz de Queiroz College of Agriculture (ESALQ), University of São Paulo, Piracicaba, SP, Brazil
[4] Food and Nutrition Research Center (NAPAN), University of São Paulo, São Paulo, SP, Brazil

***Correspondence:**
Beatriz Cordenunsi-Lysenko
hojak@usp.br

Supplementary Table 1. Macro and micronutrients analysis from experimental areas.

Macronutrients	H^+/Al^{3+}	Al^{3+}	Ca^{2+}	Mg^{2+}	K^+	P	S	C	pH		Soil layer
	cmol.dm^{-3}					mg.dm^{-3}		g.dm^{-3}	H_2O	$CaCl_2$	
**Near-NF**	2.65[a]	0.00	6.00	2.17	0.62	5.47	25.52	14.22	5.84	5.41	0-20 cm
**Distant-NF**	2.61	0.00	5.66	2.05	0.23	2.53	33.05	10.51	6.05	5.86	
**Near-NF**	2.79	0.80	2.50	1.03	0.21	2.37	44.38	7.20	5.48	4.81	20-40 cm
**Distant-NF**	2.95	0.55	1.65	1.00	0.10	2.00	105.76	5.25	5.86	5.10	
**Near-NF**	2.81	1.80	0.98	0.43	0.04	2.49	49.91	6.03	5.61	4.39	B horizon
**Distant-NF**	2.17	0.00	2.53	1.62	0.08	2.59	74.03	15.25	5.77	4.92	

Micronutrients	Fe	Cu	Mn	Zn	Textural classification	Soil layer
	mg.dm^{-3}					
**Near-NF**	155.50	7.29	15.79	1.89	loam silty clay	0-20 cm
**Distant-NF**	167.10	5.63	126.50	5.82	loam silty clay	
**Near-NF**	218.20	4.68	72.39	1.68	loam silty clay	20-40 cm
**Distant-NF**	223.60	2.85	49.16	1.71	loam silty clay	
**Near-NF**	214.50	3.25	47.12	1.68	loam	B horizon
**Distant-NF**	223.20	2.28	60.30	1.70	loam	

Values represent soil analysis from a mixed sample collected in five aleatory spots of each area. Near-NF: Near from Natural Forest area; Distant-NF: Distant from Natural forest area.

Supplementary Table 2. Foliar analysis of plant leaves from experimental areas.

Macronutrients	Mg	Ca	K	N	P	S
	g.kg^{-1}					
**Near-NF**	3.48 ± 0.36	6.57 ± 4.18	25.03 ± 4.49	24.6 ± 2.00	11.92 ± 2.05	11.57 ± 6.81
**Distant-NF**	3.52 ± 1.23	11.05 ± 4.36	23.87 ± 2.11	30.17 ± 3.47	10.04 ± 1.18	10.27 ± 4.23

Micronutrients	Fe	Cu	Mn	Zn	B
	mg.kg^{-1}				
**Near-NF**	48.83 ± 7.53	8.4 ± 2.52	579.4 ± 248.34	11.03 ± 1.85	31.44 ± 2.38
**Distant-NF**	68.73 ± 15.11	9.6 ± 3.58	622.1 ± 251.49	10.23 ± 2.12	28.75 ± 3.62

No significant differences were found on comparison between areas (Student´s T test, $p < 0.05$; $n = 3$). Near-NF: Near from Natural Forest area; Distant-NF: Distant from Natural forest area.

a

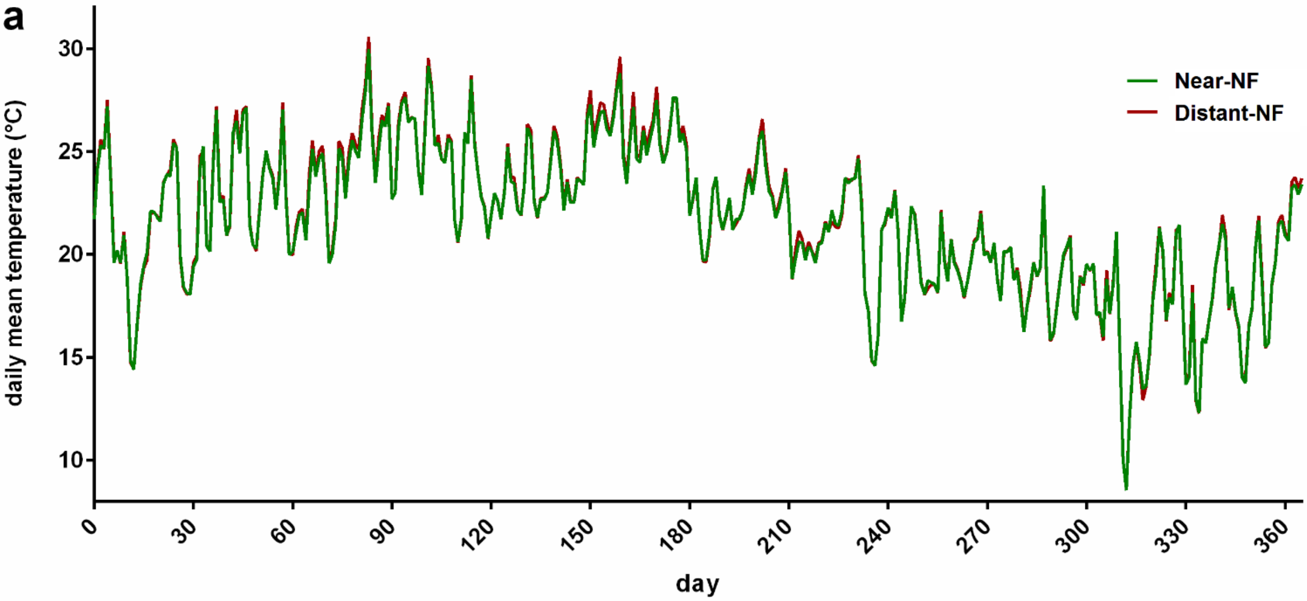

b

	Sep 2012	Oct 2012	Nov 2012	Dec 2012	Jan 2013	Feb 2013	Mar 2013	Apr 2013	May 2013	Jun 2013	Jul 2013	Aug 2013
**Near-NF**	20.68 (3.40)	22.69 (2.75)	22.70 (1.95)	25.83 (1.75)	23.39 (1.81)	25.26 (1.76)	23.58 (2.28)	22.00 (1.81)	20.01 (2.69)	19.43 (1.39)	16.73 (3.01)	17.47 (2.60)
**Distant-NF**	20.72 (3.49)	22.75 (2.82)	22.88 (2.04)	26.04 (1.82)	23.46 (1.87)	25.53 (1.91)	23.66 (2.40)	22.12 (1.86)	20.02 (2.72)	19.46 (1.39)	16.70 (3.05)	17.53 (2.62)

Supplementary Figure 1. Temperature profile from experimental areas. (a) Temperature values from areas Near from Natural Forest (Near-NF) and Distant from Natural Forest (Distant-NF) were captured every five minutes by Tinytag Plus 2 (TGP-4500, Gemini Data Loggers, Chichester, UK). **(b)** Monthly mean temperature (°C) from areas Near-NF and Distant-NF. Results represent mean (SD).

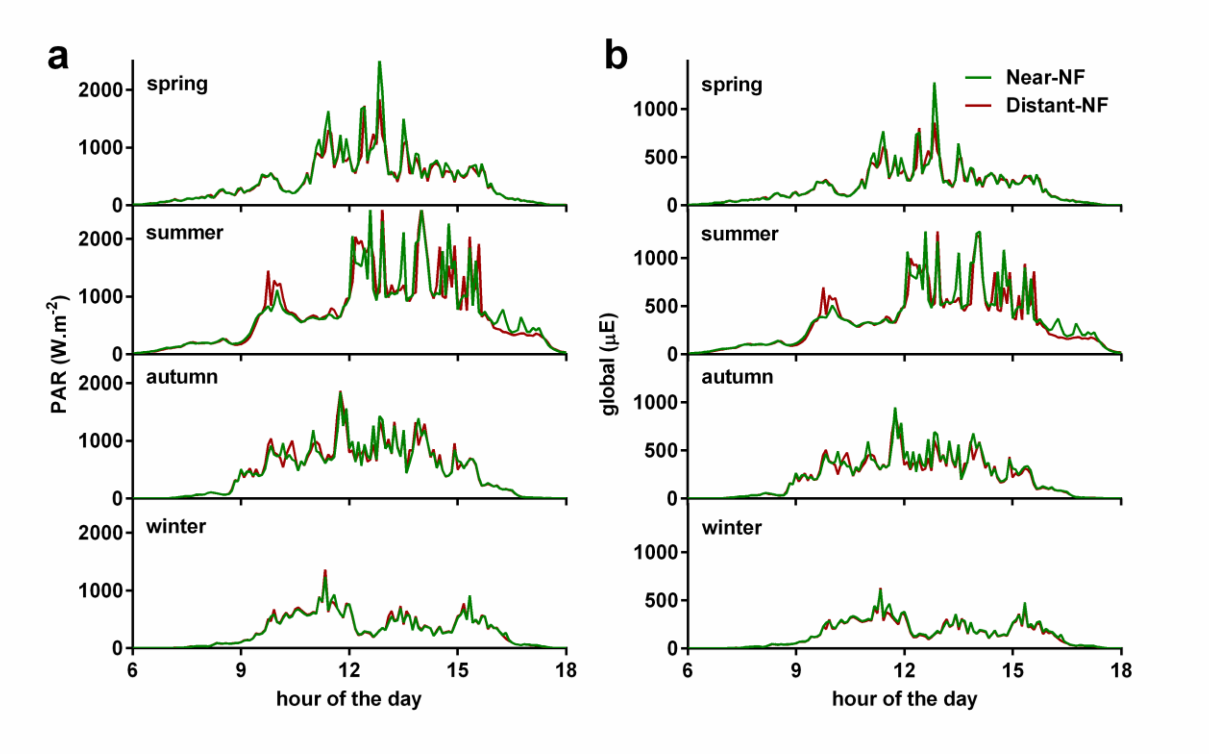

Supplementary Figure 2. Photosynthetically active (PAR) and global radiation profile from experimental areas. (a) Mean PAR and **(b)** global radiation from areas Near from Natural Forest (Near-NF) and Distant from Natural Forest (Distant-NF) were captured every minute by S-LIA-M003 and S-LIB-M003 sensors (Onset HOBO Data Loggers, Bourne, USA), respectively. Peaks and depressions indicate light variation from cloud occurrence.

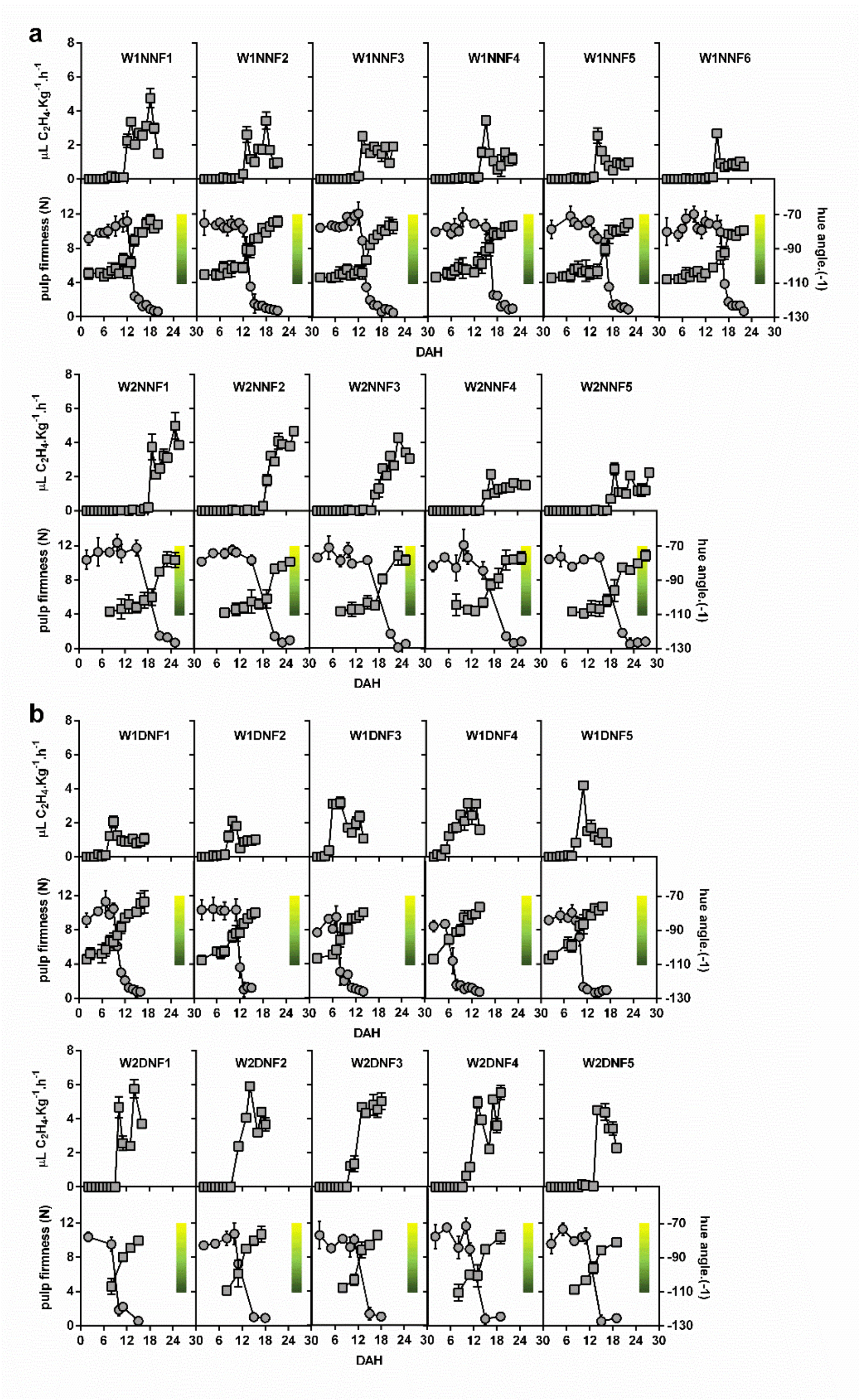

Supplementary Figure 3. Ripening parameters of fruits from plants collected on winter. Ethylene production (■) (upper graph) and peel color (■) and pulp firmness (●) (lower graph) of fruits from plants collected two times on winter (W1 and W2) and harvested from banana areas **(a)** near from natural forest (Near-NF; 11 plants) and **(b)** distant from natural forest (Distant-NF; 10 plants). DAH: days after harvest. Each graph represents values from fruits of a single plant. Group of fruits from a single plant was coded accordingly to the time of collection (W1/W2), area (Near-NF: NNF/Distant-NF: DNF) and number of plant (1–6). Each point represents the mean ± SD (n ≥ 3).

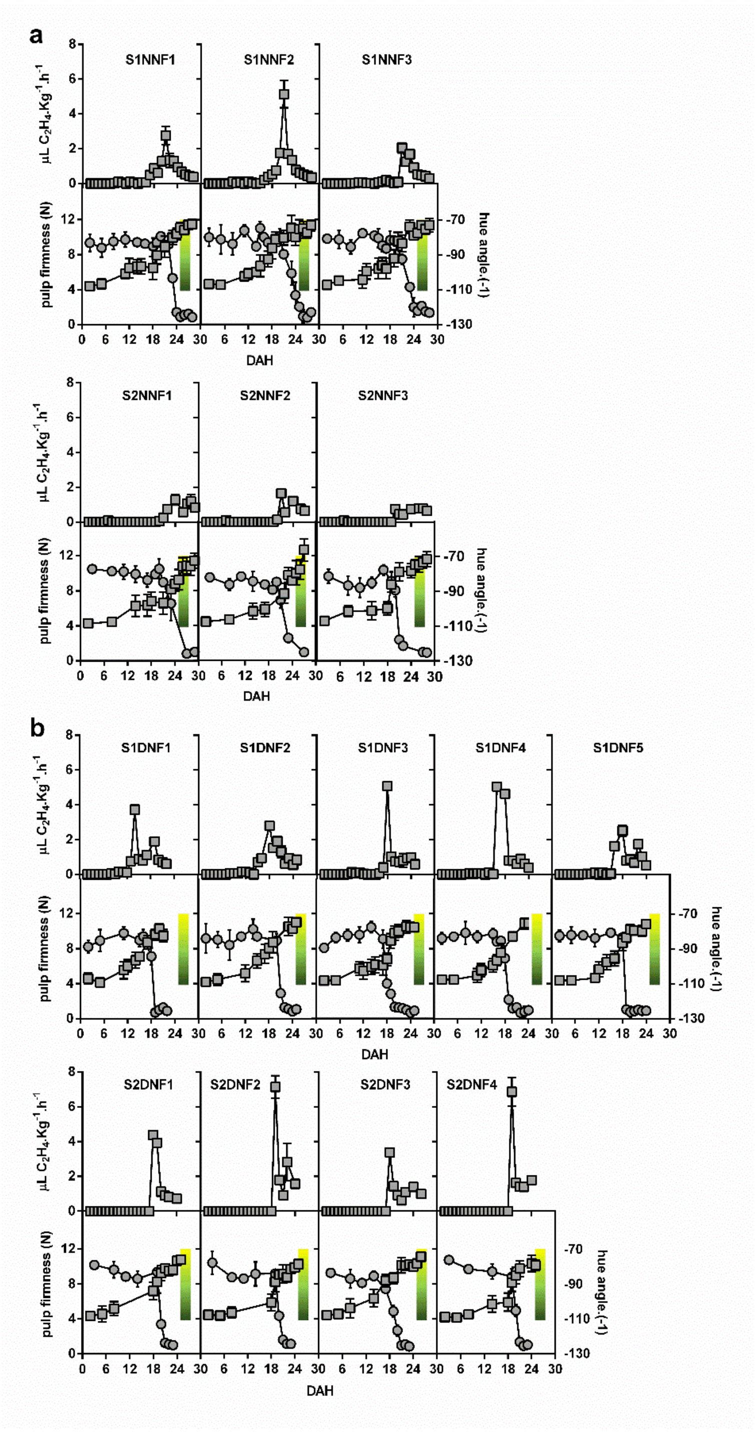

Supplementary Figure 4. Ripening parameters of fruits from plants collected on spring. Ethylene production (■) (upper graph) and peel color (■) and pulp firmness (●) (lower graph) of fruits from plants collected two times on spring (S1 and S2) and harvested from banana areas **(a)** near from natural forest (Near-NF; 6 plants) and **(b)** distant from natural forest (Distant-NF; 9 plants). DAH: days after harvest. Each graph represents values from fruits of a single plant. Group of fruits from a single plant was coded accordingly to the time of collection (S1/S2), area (Near-NF: NNF/Distant-NF: DNF) and number of plant (1–5). Each point represents the mean ± SD (n ≥ 3).

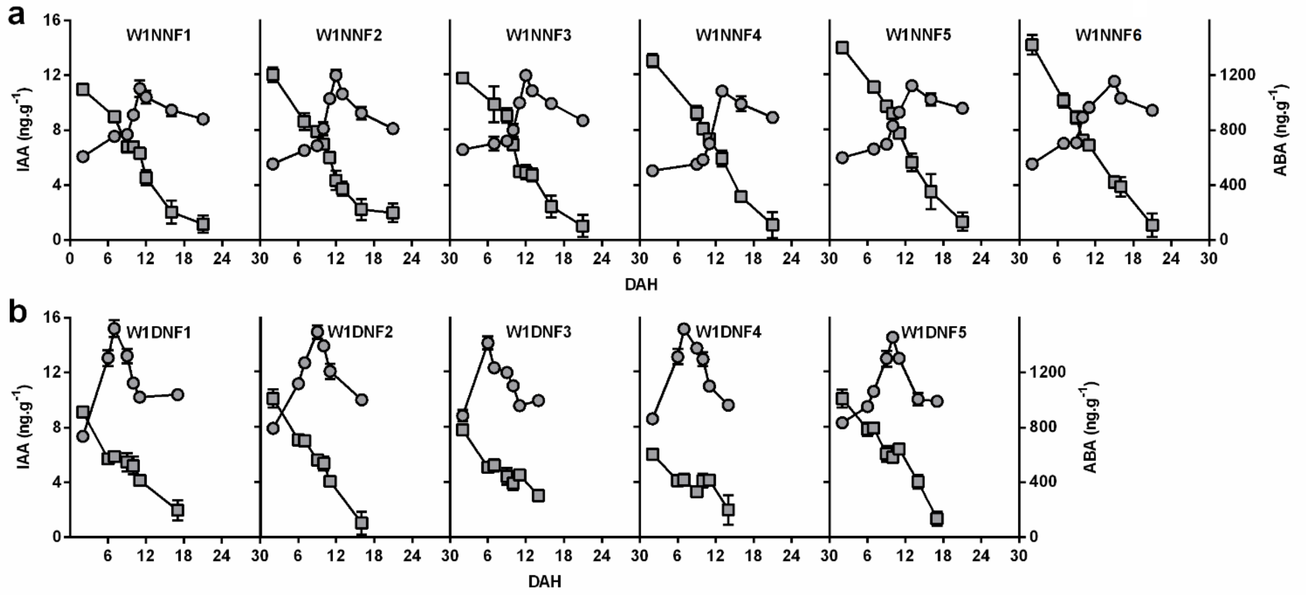

Supplementary Figure 5. Levels of free indol-3-acetic acid (IAA) (■) and abscisic acid (ABA) (●) of banana fruit pulp during ripening. Fruits were harvested from banana areas **(a)** near from natural forest (Near-NF; 5 plants) and **(b)** distant from natural forest (Distant-NF; 5 plants) collected on winter (W1). Each graph represents values from fruits of a single plant. Group of fruits from a single plant was coded accordingly to the time of collection (W1), area (Near-NF: NNF/Distant-NF: DNF) and number of plant (1–6). Each point represents the mean ± SD (n = 3).

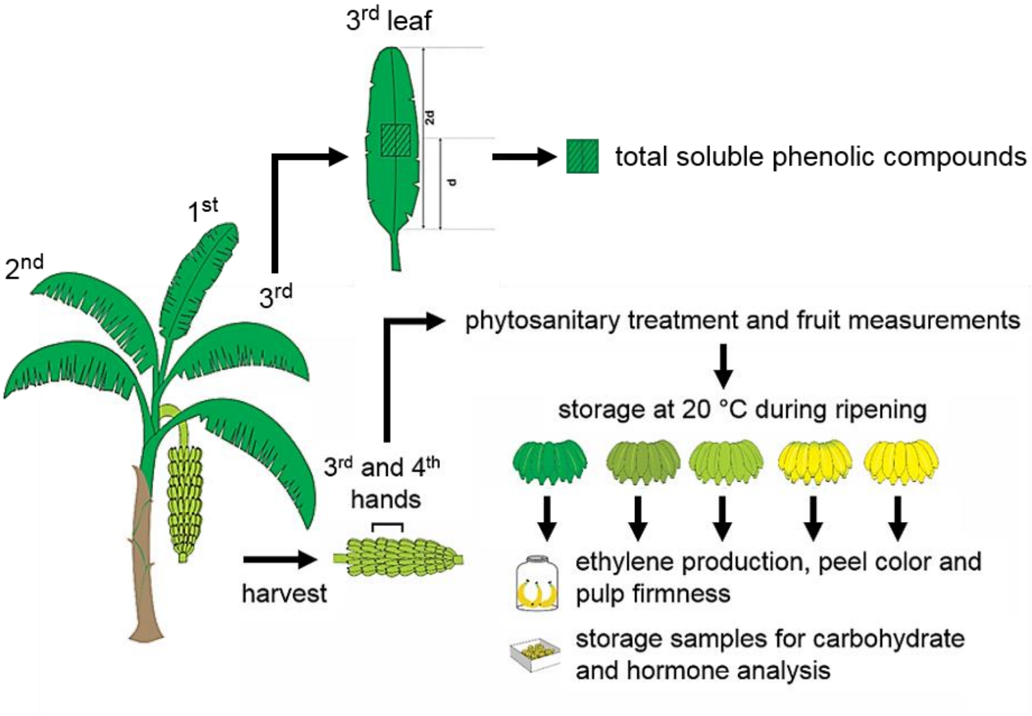

Supplementary Figure 6. Leaf and fruit sampling. Leaf sampling: the 3rd leaf starting from the top of plant was collected at the same time in which fruits were harvested. The central zone of leaf was used for total soluble phenolic analysis. Fruit sampling: after harvest at 900 degree-days, fruits from 3rd and 4th hands of bunch were selected for analysis of postharvest behavior. After phytosanitary treatment, fruits from each plant were periodically analyzed for ethylene production, peel color, pulp firmness, and pulp samples of each group were frozen at -80 °C for further carbohydrate and hormone analysis.
